# Relevance of pathogenicity prediction tools in human *RYR1* variants of unknown significance

**DOI:** 10.1038/s41598-021-82024-7

**Published:** 2021-02-09

**Authors:** Kerstin Hoppe, Karin Jurkat-Rott, Stefanie Kranepuhl, Scott Wearing, Sebastian Heiderich, Sonja Merlak, Werner Klingler

**Affiliations:** 1 Department of Anesthesiology, Intensive Care Medicine and Pain Therapie, University of Wuerzburg, Oberduerrbacher Straße 6, 97080 Wuerzburg, Germany; 2 Department of Anesthesiology, Intensive Care Medicine and Pain Therapy, University of Frankfurt, Goethe University, Theodor–Stern–Kai 7, 60590 Frankfurt, Germany; 3 Division of Experimental Anesthesiology, University Medical Center Ulm, Albert-Einstein-Allee 23, 89081 Ulm, Germany; 4 Institute of Health and Biomedical Innovation, Queensland University of Technology, 60 Musk Avenue, Kelvin Grove, 4059 Australia; 5 Department of Conservative and Rehabilitation Orthopedics, Faculty of Sport and Health Science, Technical University of Munich, Georg-Brauchle-Ring 60/62, Munich, Germany; 6 Clinic of Anesthesiology and Intensive Care Medicine, Hannover Medical School, Hannover, Germany; 7 Department of Anesthesiology, Intensive Care Medicine and Pain Therapy, SRH Clinic, Hohenzollernstraße 40, Sigmaringen, Germany

**Keywords:** Genetics, Neuroscience, Diseases, Health care, Medical research

## Abstract

Malignant hyperthermia (MH) is a pharmacogenetic disorder of skeletal muscle metabolism characterized by generalized muscle rigidity, increased body temperature, rhabdomyolysis, hyperkalemia and severe metabolic acidosis. The underlying mechanism of MH involves excessive Ca^2+^ release from myotubes via the ryanodine receptor type 1 (RYR1) and the voltage-dependent L-type calcium channel (CACNA1S). As more than 300 variants of unknown significance have been detected to date, we examined whether freely available pathogenicity prediction tools are able to detect relevant MH causing variants. In this diagnostic accuracy study, blood samples from 235 individuals with a history of a clinical malignant hyperthermia or their close relatives were genetically screened for *RYR1* variants of all 106 *RYR1* exons and additionally for known variants of *CACNA1S*. In vitro contracture tests were conducted on muscle biopsies obtained from all individuals, independently of whether a pathogenic variant, a variant of unknown significance or no variant was detected. Comparisons were made to three established bioinformatic pathogenicity detection tools to identify the clinical impact of the variants of unknown significance. All detected genetic variants were tested for pathogenicity by three in silico approaches and compared to the in vitro contracture test. Sensitivity and specificity of exon screening of all individuals listed in our MH database was analyzed. Exon screening identified 97 (41%) of the 235 individuals as carriers of pathogenic variants. Variants of unknown significance were detected in 21 individuals. Variants of unknown significance were subdivided into 19 malignant-hyperthermia-susceptible individuals and 2 non-malignant-hyperthermia-susceptible individuals. All pathogenic variants as well as the malignant-hyperthermia-suspectible variants were correctly identified by the bioinformatic prediction tools. Sensitivity of in silico approaches ranged between 0.71 and 0.98 (Polyphen 0.94 [CI 95% 0.75; 0.99]; Sift 0.98 [CI 95% 0.81; 0.99]; MutationTaster 0.92 [CI 95% 0.75; 0.99]). Specificity differed depending on the used tool (Polphen 0.98 [CI 95% 0.32; 0.99]; Sift 0.98 [CI 95% 0.32; 0.99]; MutationTaster 0.00 [CI 95% 0.00; 0.60]). All pathogenic variants and variants of unknown significance were scored as probably damaging in individuals, demonstrating a high sensitivity. Specificity was very low in one of the three tested programs. However, due to potential genotype–phenotype discordance, bioinformatic prediction tools are currently of limited value in diagnosing pathogenicity of MH-susceptible variants.

## Introduction

Malignant hyperthermia (MH) is a rare pharmacogenetic disorder, resulting in excessive Ca^2+^ release from the sarcoplasmic reticulum after activation of the abnormal ryanodine receptor (RYR1 encoded by the *RYR1* gene). In genetically predisposed individuals, activation of abnormal RYR1 can induce life-threating hypermetabolic events characterized by generalized skeletal muscle rigidity, hyperthermia, cardiac arrhythmia and serve acidosis^1–3^. Volatile anesthetics are potent activators of abnormal RYR1^1^. Moreover, rhabdomyolysis which can be caused even by non-anesthetic drugs has been reported in MH-susceptible individuals with *RYR1* variants^4^. Variants in the *CACNA1S* gene have also been identified in malignant hyperthermia susceptibility (MHS). Identification of MHS is particularly important given the high mortality rate associated with the syndrome if prompt treatment is not initiated following the onset of clinical signs.

Ca^2+^ release in muscle is mediated by direct protein–protein interaction between the voltage sensor on the plasmalemma in the region of the t-tubule (Cav1.1 encoded by the *CACNA1S* gene) and the RYR1 of the sarcoplasmic reticulum^5^. The *RYR1* gene consists of 106 exons with at least 16 non-pathogenic variants of the coding region^6,7^. Currently more than 400 malignant hyperthermia associated variants of unknown significance in the gene coding for RYR1 have been detected. To date, only about 50 variants have been proven to be pathogenic variants for MH according to the criteria of the European Malignant Hyperthermia Group (www.emhg.org)^8^. Before being classified as pathogenic, a variant must be both genetically and functionally characterized^9^. The criteria of the European Malignant Hyperthermia Group recommends that a functional assay with muscle biopsy and in vitro contracture test is required if a variant of unknown significance is detected^10,11^.

The increasing availability and decreasing cost of conducting DNA sequencing and next generation sequencing will likely result in the detection of a large number of variants of unknown significance^12^. To reveal the pathogenic potency of a detected variant of unknown significance, several in silico prediction methods have been developed. Since experimental methods are not a pragmatic approach to evaluate the large quantity of variation data currently generated, the guidelines of the American College of Medical Genetics and Genomics (ACMG) and the European Society of Human Genetics (ESHG) recommend the use of computational predictors as one of several lines of evidence for variant interpretation^13,14^. The recommendation echoes that of the joint consensus recommendation of the Association for Molecular Pathology, American Society of Clinical Oncology, and College of American Pathologists, in which the use of computational predictors have been advocated as a first line interpretation of genetic variants in cancer^15,16^. In contrast to cancer or most other genetic associated diseases, MH represents a pharmoco-genetic disorder and therefore needs application of a pharmacological trigger. Therefore such guidelines should either not be used or be used only with extreme caution for the prediction of disease-causing variants responsible for MH. Although prediction tools might offer diagnostic information in disease-causing variants, they currently should not be applied for definitive classification.

To date, several studies have investigated the predictive value of in silico approaches including SIFT, Polyphen or MutationTaster in cancer or neurodegenerative diseases^17,18^. Whether such in silico approaches might be valuable in diagnosing neuromuscular diseases remains unknown. To evaluate whether these methods may be helpful in identifying MH-susceptible individuals, we compared data from the MH center at the University of Ulm with the prediction results of the three most commonly used and freely available in silico programs: SIFT, Polyphen-2 and MutationTaster. These three in silico methods are based on different algorithm. SIFT predicts whether amino acid substitutions affect protein function, while Polyphen-2 predicts the possible impact of amnio acid substitution on the function and structure of a protein. MutationTaster, in contrast applies a combination of in silico tests. In addition to single amino acid substitution predictions, MutationTaster also determines synonymous and intronic variants, short insertion and deletion variants and even variants spanning the intron–exon borders.

## Materials and methods

### Individuals

This study is a retrospective analysis of genetic screening and in vitro muscle testing collected from 235 consecutive individuals referred to the malignant hyperthermia center (Ulm, Germany) over a 25-year period (01/1990 to 02/2015). Indications for malignant hyperthermia testing were; (1) an adverse anaesthetic event of the individual or a close relative, (2) a family–history of established MH, or (3) chronic isolated creatine kinase elevation. Patients that had only one diagnostic procedure (in vitro muscle testing or genetic screening) or more than one detected variant were excluded from this study. Written informed consent was obtained from individuals prior to their participation and all procedures were approved by the ethics committee of the University of Ulm, Germany. All methods were carried out in accordance with the guidelines of the University of Ulm Ethic Committee and the guidelines of the European Malignant Hyperthermia Group.

### Exon screening

Exon screening was performed on all individuals as described in detail previously^19,20^. Ethylendiamintetraacetate blood samples of 235 individuals were genetically screened for variants in all 106 exons of the *RYR1* gene and for known variants of *CACNA1S*^20^. DNA was extracted and amplified by polymerase chain reaction (PCR) for further analysis. PCR samples were mixed with the wild–type amplicons, denatured at 95 °C for 5 min and then cooled at room temperature to allow heteroduplexes to form^20^. Amplicons with an altered denaturing high-performance liquid chromatography (HPLC) elution profile compared to wild-type amplicons were directly sequenced with dye-terminator chemistry (Applied Biosystems)^20^.

### IVCT

In accordance with the recommendations of the European Malignant Hyperthermia Group, MHS was diagnosed using the in vitro contracture test (IVCT) as described in detail in our previous study^20–22^. Muscle samples from all 235 individuals were tested, irrespective of whether a pathogenic, benign, variant of unknown significance or no variant was detected. In brief, the in vitro contracture test determines the contractile sensitivity of surgically excised muscle specimens to halothane and caffeine^20^. Biopsies were taken from the vastus lateralis muscle under regional anesthesia. Basal tension and the twitch response to supramaximal electrical stimulation (30–70 V, 0.2 Hz, 1 ms) was recorded with a force transducer^20^. Muscle bundles from malignant hyperthermia susceptible individuals (MHS) have lower contractile thresholds to caffeine and halothane than non- malignant hyperthermia susceptible individuals (MHN)^20^. A positive result for malignant hyperthermia susceptibility was defined as a contracture force of ≥ 2 mN at a caffeine concentration ≤ 2 mM and a halothane concentration ≤ 2% v/v^21^. A major limitation by diagnosing malignant hyperthermia using the IVCT is that it results in a categorical classification rather than individual interpretation of continuous contracture data^20^.

### In silico approach

All detected pathogenic variants and variants of unknown significance, including malignant hyperthermia susceptible and non-malignant-hyperthermia-susceptible as determined by the in vitro contracture test, were analyzed by application of three in silico prediction programs. Additionally four previously characterized benign variants (c.5360C < T, synonymous; c.6178G < T, synonymous; c.10747G > C, non-coding; c.11266C > G, synonymous) were tested. In this study Sorting Intolerant from Tolerant (SIFT) (http://sift.jcvi.org), Polymorphism Phenotyping vs. 2 (Polyphen-2) (http://genetics.bwh.harvard.edu/pph2/) [HumDiv dataset] and MutationTaster (http://www.mutationtaster.org/) were applied.

SIFT (sorting intolerant from tolerant) predicts whether an amino acid substitution affects protein function by comparing amino acid alignments from related sequences to calculate a “swift score”. A score between 0.00 and 0.05 is classified as “damaging” while a score between 0.05 and 1.00 is classified as “tolerated”.

Polyphen-2 (Polymorphism Phenotyping vs.-2) predicts the possible impact of amino acid substitution on the structure and function of a human protein. Variants are classified as “benign”, “possibly damaging” or “probably damaging” with scores ranging between 0.0 (benign) and 1.0 (probably damaging).

MutationTaster combines a battery of in silico tests to predict the pathogenicity of variants of unknown significane at the protein and DNA level. Thus, MutationTaster is not limited to substitutions of single amino acids and can determine synonymous and intronic variants, short insertion or deletion variants and variants spanning intron-exons borders.

### Statistical analysis

With an estimated prevalence of disease of 80%, sample size calculation (PASS software) revealed that 39 individuals were required to detect a difference of 20% between the in vitro contracture test and the in silico approaches at a significance level of 0.05 and with 80% power. A confusion matrix was used to evaluate the performance of all in silico approaches tested in this study. To this end, true positive was identified when the in vitro contracture test result corresponded with a positive result (pathologic or damaging) of the in silico method. A true negative, in contrast, was identified when the in vitro contracture test result corresponded with a negative result (neutral or benign) of the in silico method. Consequently, we calculated, (1) the sensitivity (proportion of positively identified cases with a true pathogenic variant) and (2) the specificity (proportion of positively identified cases with a true neutral variant) of all three in silico approaches. Positive (3) and negative (4) predictive values were also calculated for each test. Lower (5) and upper (6) 95% confidence interval were also calculated for all tests. Means and standard deviations were calculated for score values (pathogenic, benign and variants of unknown significance) obtained from all three in silico approaches. A alpha level of 0.05 was used for all tests of significance. StatXaxt (Version 5, Cytel Software, Cambridge, MA) and Excel (Version 2010, Microsoft, Redmon, Washington, USA) were used for all statistical procedures.

The following outcome variables were calculated:

1. Sensitivity: True positive/True positive + false negative
2. Specificity: True negative/True negative + false positive
3. Positive predictive value: True positive / True positive + false positive
4. Negative predictive value: True negative / True negative + false negative
5. Accuracy: True positives + True negatives / True positives + True negatives + False positives + False negatives
6. Lower 95% confidence interval: X−1.96 √ [X * (1−X)/n];
7. Upper 95% conficdence interval: X + 1.96 √ [X * (1−X)/n];

where X represents the sensitivity, specificity, positive predictive value, negative predictive value.

## Results

### Characterization of individuals

**Figure 1:**
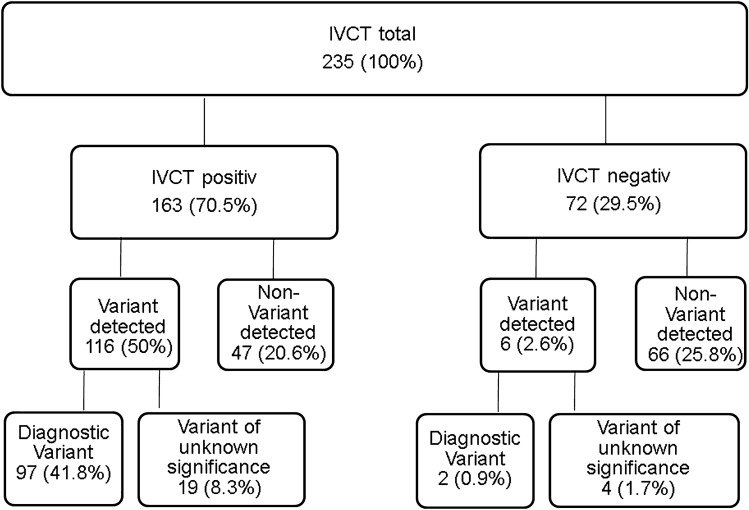
Results of all tested individuals. A total of 235 individuals with a history of clinical malignant hyperthermia or close relatives were functionally analysed by the in-vitro contracture test (IVCT) and genetically screened. The detected variants were divided into known pathogenic variants and variants of unknown significance. For classification as being pathogenic, each variant has to be fully characterized at the genetic level, including co-segregation of the variant with the disease in the family affected. Moreover, functional assays have to show that a variant in the *RyR1* or *CACNA1S* gene results an effect consistent with the MH phenotype. All other variants are classified as variant of unknown significance.

In total, 235 [93 female; 142 male] [median age: 35 (20;51)] individuals underwent in vitro contracture testing during the 25-year study period. According to the in vitro contracture testing protocol of the European Malignant Hyperthermia Group, 163 were classified as malignant hyperthermia susceptible and 72 as non-malignant hyperthermia susceptible. Exon screening identified 122 individuals with a genetic variant. All of them were identified in the *RYR1* gene. 99 of these Individuals possessed a known *RYR1* pathogenic variant and 23 a variant of unknown significance [Fig. 1]. All variants that were detected are shown in Supplement 1 + 2. Due to missing data from the in vitro contracture test, threshold levels and the results of the exposure to other trigger substance were used for diagnosis in sixteen individuals, in accordance to the European Malignant Hyperthermia Group guidelines.

### Comparison of in vitro contracture testing versus exon screening

Of the 122 individuals with an *RYR1* variant (99 with diagnostic and 23 with a variant of unknown significance), the in vitro contracture test classified 116 as malignant hyperthermia susceptible, while 6 were classified as non-malignant hyperthermia susceptible. Two of the 6 non-malignant hyperthermia suceptible individuals possessed a pathogenic variant. In 113 individuals no genetic variant was detected. 66 of these individuals were classified as non-malignant hyperthermia susceptible by applying the in vitro contracture test. However, 47 individuals were classified as malignant hyperthermia susceptible (37 were malignant hyperthermia susceptible to both compounds (MHShc; 2 were malignant hyperthermia susceptible to caffeine (MHSc); 8 were malignant hyperthermia susceptible to halothane (MHSh)). Malignant hyperthermia susceptible in vitro contracture test results were significantly different compared to non-malignant hyperthermia susceptible individuals [Supplement data].

### In silico analysis of variants on unknown significance

Each of the variants of unknown significance were genetically analyzed and categorized as either a pathogenic variant or a variant of unknown significance. Additionally, muscle specimen was tested using the in vitro contracture test. Some individuals were classified as malignant hyperthermia susceptible due to a positive result in the in-vitro contracture test without the detection of a genetic variant.

All pathogenic and all variants of unknown significance were subsequently tested with SIFT, Polyphen-2 and MutationTaster. All three in silico analysis programs had high sensitivity [Polyphen 0.94 CI 0.75; 0.99; SIFT 0.98 CI 0.81; 0.99; MutationTaster 0.91 CI 0.71; 0.97]. Sift correctly identified all 27 tested variants of unknown significance as “not tolerated”, which means that the tested variant was disease causing. MutationTaster correctly identified 25 of the 27 variants as disease causing. However, two were categorized as a “polymorphism” by MutationTaster, which does not exclude the variant as disease causing. Application of Polyphen2 detected three variants (one pathogenic variant and all malignant hyperthermia susceptible) as possibly rather than probably damaging and one as benign [Tables 1 and 2].

**Table 1:** Sensitivity and Specificity of in silico approaches.

(1)	Genetic screening	Lower 95% confidence	Upper 95% confidence
Sensitivity	0.712	0.63	0.77
Specificity	0.91	0.82	0.96
Positive predictive value	0.95	0.89	0.98
Negative predictive value	0.58	0.48	0.67

(2)	Polyphen	Lower 95% confidence	Upper 95% confidence
Sensitivity	0.94	0.75	0.99
Specificity	0.98	0.32	0.99
Positive predictive value	0.99	0.82	0.99
Negative predictive value	0.75	0.22	0.95

(3)	Sift	Lower 95% confidence	Upper 95% confidence
Sensitivity	0.98	0.81	0.99
Specificity	0.98	0.32	0.99
Positive predictive value	0.99	0.83	0.99
Negative predictive value	0.9	0.28	0.95

(4)	Mutation taster	Lower 95% confidence	Upper 95% confidence
Sensitivity	0.91	0.71	0.97
Specificity	0.01	0.005	0.55
Positive predictive value	0.85	0.65	0.94
Negative predictive value	0.02	0.01	0.76

**Table 2:** In silico approach analysis (Polyphen-2, Sift, MutationTaster).

	Mutation	dbSNP	Result polyphen	Score	Result Sift	Score	Result Mutation taster	Score
**A**								
c.130C > T	R44C	rs 193922748	Probably damaging	1.0	Not tolerated	0.94	Disease causing	180
c.742G > A	G248R	rs 1801086	Probably damaging	0.999	Not tolerated	0.96	Disease causing	125
c.1021G > A	G341R	rs 28933997	Probably damaging	0.996	Not tolerated	0.96	Disease causing	125
c.1201C > T	R401C	rs 193922765	Probably damaging	0.998	Not tolerated	0.96	Disease causing	180
c.1840C > T	R614C	rs 118192172	Probably damaging	1.0	Not tolerated	0.96	Disease causing	180
c.1841G > T	R614L	rs 193922772	Probably damaging	1.0	Not tolerated	0.96	Disease causing	102
c.5000G > A	R1667H	rs 138978909	Probably damaging	0.998	Not tolerated	1.0	Polymorphism	29
c.6487C > T	R2163C	rs 28933998	Probably damaging	1.0	Not tolerated	0.90	Disease causing	180
c.6488G > A	R2163H	rs 28933999	Probably damaging	1.0	Not tolerated	0.90	Disease causing	29
c.6617C > T	T2206M	rs 289934000	Probably damaging	1.0	Not tolerated	0.90	Disease causing	81
c.6617C > G	T2206R	rs 118192177	Probably damaging	1.0	Not tolerated	0.90	Disease causing	71
c.7007G > A	R2336H	rs 112563513	Probably damaging	0.997	Not tolerated	0.90	Disease causing	29
c.7124G > C	G2375A	rs 193922807	Benigne	0.022	Not tolerated	0.88	Disease causing	60
c.7300G > A	G2434R	rs 121918593	Probably damaging	1.0	Not tolerated	0.90	Disease causing	125
c.7360C > T	R2454C	rs 193922816	Probably damaging	1.0	Not tolerated	0.90	Disease causing	180
c.7361G > A	R2454H	rs 118192122	Probably damaging	0.998	Not tolerated	0.90	Disease causing	29
c.14497C > T	H4833Y	rs 193922876	Probably damaging	0.997	Not tolerated	0.98	Disease causing	83
**B**								
c.520C > T	R174W	rs 772226819	Probably damaging	1.0	Not tolerated	1.0	Polymorphism	101
c.1024G > A	E342K	–	Probably damaging	1.0	Not tolerated	0.96	Disease causing	56
c.3257G > A	R1086H	rs 1800559	Probably damaging	1.0	Not tolerated	0.98	Disease causing	29
c.7025A > G	N2342S	rs 147213895	possibly damaging	0.857	Not tolerated	0.90	Disease causing	46
c.7073 T > C	I2358T	–	Probably damaging	0.857	Not tolerated	0.88	Disease causing	89
c.7355G > C	R2452P	rs 193922815	Probably damaging	0.998	Not tolerated	0.96	Disease causing	103
c.10616G > A	R3539H	rs 143987857	Probably damaging	0.999	Not tolerated	0.90	Disease causing	29
c.11315G > A	R3772Q	rs 193922839	Probably damaging	0.999	Not tolerated	0.88	Disease causing	43
c.11723A > T	N3908I	–	Probably damaging	0.991	Not tolerated	0.88	Disease causing	149
c.14928C > G	F4976L	rs 368874586	Probably damaging	0.982	Not tolerated	0.88	Disease causing	22
**C**								
c.5360C > T		rs 34934920	Benigne	0.003	Tolerated	0.07	Polymorphism	98
c. 6178G > T		rs 35364374	Benigne	0.436	Tolerated	0.08	Disease causing	–
c.10747G > C		rs 55876273	Benigne	0.182	Tolerated	0.39	Disease causing	81
c.11266C > G		rs 4802584	Benigne	0.1	Tolerated	–	Disease causing	125

(A) Diagnostic variants listed on the European Malignant Hyperthermia Group website, (B) Variants of unknown significance, (C) Four added benign variants.

While two of the three in silico approaches correctly detected benign variants of unknown significance, MutationTaster suggested three as disease causing and one as a polymorphism.

For 13 of the variants, minor allele frequencies were found using GenomAD. Eight of the variants were pathogenic variants (c.130C > T, p.Arg44Cys; c.742G > A, p.Gly248Arg; c.1841G > T, p.Arg614Leu; c.6617C > T, p.Thr2206Met; c.6614C > G, p.Thr2206Arg; c.7300G > A, p.Gly2434Arg; c.7360C > T, p.Arg2454Cys; c.7361G > A, p.Arg2554His) and four were variants of unknown significance (c.7025A > G, p.Asn2342Ser; c.10616G > A, p.Arg3539His; c.11315G > A, p.Arg3767Pro; c.14928C > G, p.Phe4976Leu) [Table 3].

**Table 3:** Minor allele frequency for pathogenic and variants of unknown significance based on the ExAc.

	Variant	Genomic cordinates	Overlapping transcripts	dbSNP	Minor allele frequency genomAD
c.130C > T	R44C	chr19:38440829	ENST00000355481.8 ENST00000359596.8 LRG_766t1.1	rs 193922748	< 0.01
c.742G > A	G248R	chr19:38446710	ENST00000355481.8 ENST00000359596.8 LRG_766t1.1	rs 1801086	< 0.01
c.1021G > A, c. 1021G > C	G341R	chr19:38448712	ENST00000355481.8 ENST00000359596.8 ENST00000594335.5 LRG_766t1.1	rs 121918592	–
c.1201C > T	R401C	chr19:38451842	ENST00000355481.8 ENST00000359596.8 ENST00000594335.5 LRG_766t1.1	rs 193922764	< 0.01
c.1840C > T	R614C	chr19:38457545	ENST00000355481.8 ENST00000359596.8 ENST00000594335.5 LRG_766t1.1	–	–
c.1841G > T	R614L	chr19:38948185	ENST00000355481.8 ENST00000359596.8 ENST00000594335.5 LRG_766t1.1	rs 118192172	< 0.01
c.5000G > A	R1667H	chr19:38485655	ENST00000355481.8 ENST00000359596.8 ENST00000594335.5 LRG_766t1.1	rs 138998909	< 0.01
c.6487C > T	R2163C	chr19:38494564	ENST00000355481.8 ENST00000359596.8 ENST00000594335.5 LRG_766t1.1	rs 118192175	–
c.6488G > A	R2163H	chr19:38495565	ENST00000355481.8 ENST00000359596.8 ENST00000594335.5 LRG_766t1.1	rs 118192163	–
c.6617C > T	T2206M	chr19:38496278	ENST00000561409.1 ENST00000361243.6 ENST00000394518.7 ENST00000559362.5	rs 141646642	< 0.01
c.6617C > G	T2206R	chr19:38496283	ENST00000355481.8 ENST00000359596.8 ENST00000594335.5 LRG_766t1.1	rs 118192177	< 0.01
c.7007G > A	R2336H	chr19:38499223	ENST00000355481.8 ENST00000359596.8 ENST00000594335.5 LRG_766t1.1	rs 112563513	–
c.7124G > C	G2375A	chr19:38499731	ENST00000355481.8 ENST00000359596.8 ENST00000594335.5 LRG_766t1.1	rs 193922807	–
c.7300G > A	G2434R	chr19:38499993	ENST00000355481.8 ENST00000359596.8 ENST00000594335.5 LRG_766t1.1	rs 121918593	< 0.01
c.7360C > T	R2454C	chr19:38500642	ENST00000355481.8 ENST00000359596.8 ENST00000594335.5 LRG_766t1.1	rs 193922816	< 0.01
c.7361G > A	R2454H	chr19:38580114	ENST00000355481.8 ENST00000359596.8 ENST00000594335.5 LRG_766t1.1	rs 118192122	< 0.01
c.14497C > T	H4833Y	chr19:38948185	ENST00000355481.8 ENST00000359596.8 ENST00000594335.5 LRG_766t1.1	rs 193922876	–

	Variant	Genomic cordinates	Transcripts	dbSNP	Minor allele frequency genomAD
c.520C > T	R174W	chr1:201091993	–	–	–
c.1024G > A	E342K	chr19:11110735	–	–	–
c.3257G > A	R1086H	chr1:201060815	ENST00000362061.4 ENST00000367338.7	rs 1800559	–
c.7025A > G	N2342S	chr19:38499241	ENST00000355481.8 ENST00000359596.8 ENST00000594335.5 LRG_766t1.1	rs 147213895	< 0.01
c.7073 T > C	I2358T	chr19:38499680	–	–	–
c.7355G > C	R2452P	chr19:38500637	ENST00000355481.8 ENST00000359596.8 ENST00000594335.5 LRG_766t1.1	rs 193922815	–
c.10616G > A	R3539H	chr19:38525492	ENST00000355481.8 ENST00000359596.8 LRG_766t1.1 ENST00000599547.5 ENST00000594335.5	rs 143987857	< 0.01
c.11315G > A	R3772Q	chr19:38534775	ENST00000355481.8 ENST00000359596.8 ENST00000593322.1 ENST00000596431.5 ENST00000599547.5 ENST00000601514.5 LRG_766t1.1 ENST00000594335.5	rs 193922839	< 0.01
c.11723A > T	N3908I	–	–	–	–
c.14928C > G	F4976L	chr19:38586150	ENST00000672647.1 ENST00000675628.1 ENST00000676181.1 ENST00000676363.1 ENST00000309041.12 ENST00000547691.8 ENST00000552810.6 ENST00000673058.2 ENST00000674971.1	rs 369874586	< 0.01

## Discussion

Until now only about 48 variants of the RyR1 have been described as pathogenic for malignant hyperthermia and/or central core disease^23^www.emhg.org. For classification as being pathogenic each variant has to be fully characterized at the genetic level, including co-segregation of the variant with the disease in the affected family. Moreover, functional assays have to show abnormal calcium release when compared to normal (wild-type) RyR1^24^www.emhg.org. For the other roughly 400 known *RYR1* variants, functional data are still necessary for proof of pathogenicity. Several bioinformatic tools have been developed to aid in the prediction of pathogenicity of variants of unknown significance. However, bioinformatic tools are currently not approved for clinical diagnosis of malignant hyperthermia.

99 of the tested individuals in this study possessed a known RyR1 pathogenic variant. In accordance to previously published data, the IVCT identified 97 of these individuals as malignant hyperthermia susceptible. However, two individuals possessed a pathogenic variant but were not identified by the in vitro contracture test. Some variants (e.g. 1021G > A, 1840C > T and 7300G > A) have been reported to show phenotype/genotype discordance which may simply reflect the relative mutation prevalence^25^. Otherwise, some variants were reported to result in weaker contractures and were therefore expected to show higher discordance rates than those associated with more severe contracture phenotypes^25^. Thus all three mutations have been shown to be associated with weaker contractions compared to 487C > T, 648C > T, 6488G > A and 7304G > A by analysis of channel mutants in vitro and by comparative analysis of IVCT data in mutation carriers^25–28^.

SIFT and Polyphen-2 are the most evaluated methods to predict disease phenotype. Both algorithms are primarily based on the quantification of the constraint on the affected residue from a multiple-sequence alignment^29–31^. However, an amino acid which is not present at the substitution site in the multiple alignments can be replaced by an amino acid with the similar charge and hydrophobicity and be predicted as tolerated^29–33^. SIFT relies on mere sequence homology and is most accurate if alignments consists on mere orthologous sequences^35,36^. Polyphen, in contrast, uses UniProt entries to predict whether an amino acid substitution appears within an important structural or functional site of the protein^36^. Notably, benchmark analysis revealed that specificity and sensitivity vary significantly depending on the algorithm used. In accordance with previous studies involving *RYR1* and other genes, SIFT and Polyphen-2 were found to have a high sensitivity for detecting malignant hyperthermia. In accordance with Schiemann et al., we also found the Polyphen-2 approach had a relatively high specifity. Interestingly, both approaches were suggested as being more accurate at predicting loss of function than gain of function mutations^36^.

Finally, we tested MutationTaster in predicting pathogenicity in *RYR1*. MutationTaster has been suggested to have greater sensitivity and specificity than earlier in silico approaches^32–34^. Our data tend to confirm these results with sensitivity ranging between 0.92 and 1.0. The heightened sensitivity of MutationTaster may reflect the way which it considers only the loss or decreased strength of splice sites at existing intron–exon borders. MutationTaster automatically detects and categorizes confirmed polymorphisms and known disease variants, which has been suggested to yield a false positive rate of 1% for homozygous alterations. Interestingly, our data tends to contradict this suggestion. None of the four variants previously characterized as benign were identified correctly by MutationTaster. MutationTaster is restricted in characterizing non-exonic alterations and is unable to analyze alterations spanning an intron–exon border which may partly explain the results of this study^33^.

However, penetrance of malignant hyperthermia is currently under investigation, since there is a clear discordance between genetic prevalence and clinical frequency of malignant hyperthermia. Risk factors for the development of a clinical event include gender (male), age (youth), lack of temperature monitoring, a combination of volatile anesthetics or succinylcholine, hyper-CKemia and most notably, the presence of diagnostic variants or presence of variants of unknown significance.

Finally, malignant hyperthermia susceptibility is unique in that a trigger compound is required and no consistent phenotype can be detected without a trigger compound. Clinical events may be due to incomplete penetrance and variable expressivity and may result in malignant hyperthermia crises even after safe exposure to a trigger anesthetic^37^.

Although the American and European guidelines currently recommend that the in vitro contracture test should be used to diagnose malignant hyperthermia it is possible that a positive contracture test might be associated with several deficiencies in phenotyping. The IVCT also suffers from poor to unknown inter-laboratory reliability, and an absence of data on the between-day repeatability. Similarly as the number of individuals with a neuromuscular disease that is not associated with malignant hyperthermia but still return a positive in vitro contracture test result is unknown, the true specificity of the test remains undetermined but is potentially knowable. Moreover, the penetrance and expressivity of the in vitro contracture test trait are unknown^37^. New diagnostic tests are frequently evaluated against “gold standards” which are assumed to classify individuals with unerring accuracy according to presence or absence of disease. Practically, however these “gold standard” tests are rarely perfect predictors of disease and tend to misclassify a small number of individuals^38^. When an imperfect “gold standard” is used to determine disease status this might subsequently introduce bias into measurements of test performance. For instance, the sensitivity and specificity of a new test will be underestimated, if the new test and imperfect standard test are conditionally independent (i.e. do not err on the same individuals)^38^.

**Figure 2:**
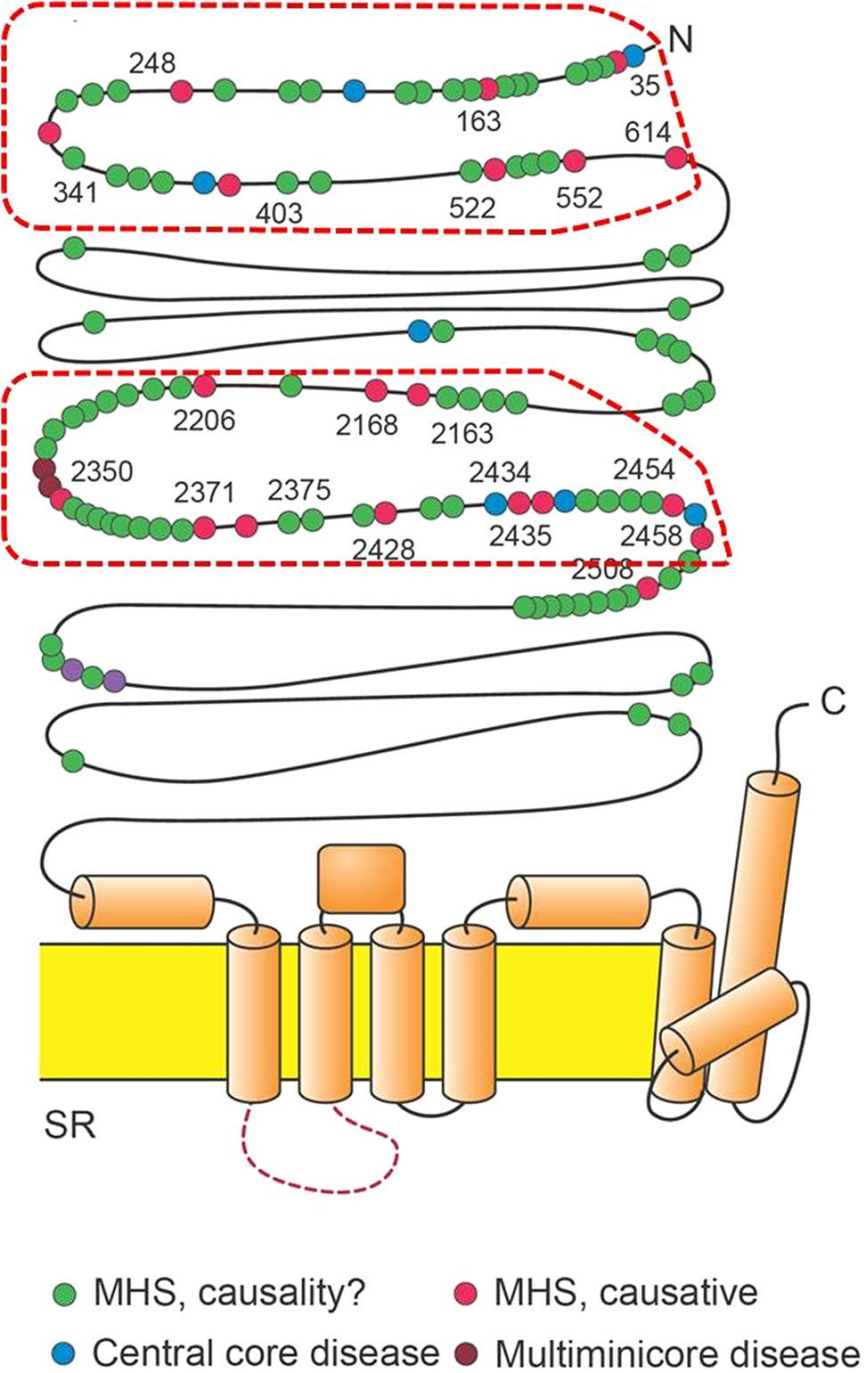
Schematic presentation of the *RYR1* gene. Regions with the majority of the pathogenic variants were marked with a red line. Mutations also have been found in the transmembrane domains. In contrast to the mutations in the N-terminal end or in the mid-portion of the receptor, these mutations might only influence the binding of modifying ligands but also directly affect calcium pore flux. Central core disease and multi minicore disease are neuro-muscular disorder which are due to its gene proximity and overlap often associated with malignant hyperthermia.

In the current study, the majority of pathogenic *RYR1* variants appeared clustered on the N-terminal amino acid residues, i.e. RYR1 AA 35-614, and the centrally located residues, i.e. AA 2163-2458 [Fig. 2]. All of the *RYR1* mutations result in amino acid substitutions in the myoplasmic portion of the protein, with the exception of one mutation in the C-terminus which resides in the transmembrane region. Functional analysis showed inconsistent abnormalities. RYR1 channel kinetics might not simply be changed to be over-reactive to volatile anesthetics, but changes in inactivation kinetics and sensitivity to physiologically channel modifiers such as lipids, ATP and Mg^2+^ also discussed within the literature might occur. Some variants in the C-terminal region of RyR1 protein are associated with excitation–contraction uncoupling or a partially depleted sacroplasmic reticulum through a constant Ca^2+^ leak. Long-term myoplasmic Ca^2+^ overload has been associated with mitochondrial damage resulting in core myopathies. For some RYR1 mutations lower Ca^2+^ peak levels and lower sensitivity to Ca^2+^ releasing drugs have been described. Currently, there are insufficient genotype–phenotype associations, to make a definitive statement about clinical risk based on the variant type alone^39,40^. Therefore evaluation of “functional significance” is not trivial for several *RYR1* mutations.

## Conclusion

Sensitivity and specificity of in silico approaches predict potential pathogenicity of variants of unknown significance in malignant hyperthermia is improving. However, based on the findings of the current study, we still recommend following the guidelines of the European Malignant Hyperthermia Group which advises to conduct a further functional test especially if the detected variants is not diagnostic. In vitro contracture testing has been shown to have a high sensitivity (97–99%) and acceptable specificity (approximately 70%) which may be increased to 94% by using two trigger compounds (halothane and caffeine)^20^. Such values might also be achieved by the application of the combination of bioinformatic based prediction approaches. However, variants of *RYR1* or *CACNA1S* are only detectable in about 50–70% of the malignant hyperthermia families^41,42^. Five other loci have been identified by linkage analysis^41,42^. Since the detection of further loci cannot be excluded, functional approaches remain obligatory.

## Supplementary information

Supplementary Information 1.

Supplementary Information 2.

Supplementary captions.
