# Supplementary material for: Relevance of pathogenicity prediction tools in human RYR1 variants of unknown significance
Source: Sci Rep. 2021 Feb 9;11:3445. doi: 10.1038/s41598-021-82024-7 (PMC7873245; doi:10.1038/s41598-021-82024-7)
Supplement: Supplementary file 1 — Supplementary Information 1. [file 41598_2021_82024_MOESM1_ESM.pdf]

				Mutation positiv individuals				*Mutation negativ individuals*			
	**Mutation**	**Code-Nr.**	**IVCT(n)**	**MHS(n)**	**MHSc(n)**	**MHSh(n)**	**MHN(n)**	**MHS(n)**	**MHSc(n)**	**MHSh(n)**	**MHN(n)**
**c.1840C>T**	R614C	MH 1	2	1				1			
	R614C	MH 17	2	2							
	R614C	MH 18	3	1							2
	R614C	MH 31 ATA	3	1							2
	R614C	MH 49	1	1							
	R614C	MH 62	2	2							
	R614C	MH 67	2	1					1		
	R614C	MH 72	1			1					
	R614C	MH 76	**4**	2		**1**					1
	R614C	MH 86	10	7					1		2
	R614C	MH 119	**5**	**3**							2
	R614C	MH 165	4			1		2			1
	R614C	MH 181 DIST 3	3	1							2
	R614C	MH 249	1	1							
			**43**	**23**	**0**	**3**	**0**	**3**	**2**	**0**	**12**
**c.1841G>T**	R614L	MH 38	**1**	**1**	**0**	**0**	**0**	**0**	**0**	**0**	**0**
**c.7300G>A**	G2434R	MH 8	2	1							1
	G2434R	MH 14	8	3			1	3			1
	G2434R	MH 22	1			1					
	G2434R	MH 29 CAT	13	11		1					1
	G2434R	MH 90	7					2			5
	G2434R	MH 102	12	7				1		3	1
	G2434R	MH 106	1	1							
	G2434R	MH 109	2	1							1
	G2434R	MH 129	3	2				1			
	G2434R	MH 191	4	3							1
	G2434R	MH 194	5	2						1	2
	G2434R	MH 199	2	2							
	G2434R	MH 210	3	1							2
	G2434R	MH 215	1	1							
	G2434R	MH 241	3	2				1			
			**67**	**37**	**0**	**2**	**1**	**8**	**0**	**4**	**15**
**c.7007G>A**	R2336H	MH 71	10	4							6
	R2336H	MH 52	1					1			
	R2336H	MH 85	10	3				1		2	4
			**21**	**7**	**0**	**0**	**0**	**2**	**0**	**2**	**10**
**c.5000G>A**	R1667H	MH 90	**7**	**2**	**0**	**0**	**0**	**0**	**0**	**0**	**5**
**c.130C>T**	R44C	MH 3	**9**	**4**	**0**	**0**	**0**	**5**	**0**	**0**	**0**
**c.6617C>T**	T2206M	MH 56	4	3						1	
	T2206M	MH 83	**7**			**1**		2	1		**3**
	T2206M	MH 126	2	2							
	T2206M	MH 141	1	1							
			**14**	**6**	**0**	**1**	**0**	**2**	**1**	**1**	**3**
**c.6617C>G**	T2206R	MH 33	**7**	**2**	**0**	**0**	**0**	**1**	**0**	**1**	**3**
**c.14497C>T**	H4833Y	MH 2	**7**	**1**				**1**	**1**		**4**
**c.7360C>T**	R2454C	MH 5	1	1							
	R4945X	MH 21	2	1			1				
			**3**	**2**	**0**	**0**	**1**	**0**	**0**	**0**	**0**
**c.11315G>A**	R3772Q	MH 42	**1**	**1**	**0**	**0**	**0**	**0**	**0**	**0**	**0**
**c.7025A>G**	N2342S	MH 85	**9**	**1**	**0**	**1**	**2**	**3**	**0**	**0**	**2**
**c.7124G>C**	G2375A	MH 51	**2**	**1**				**1**			
**c.6487C>T**	R2163C	MH 63	**2**	**1**	**0**	**0**	**0**	**0**	**0**	**0**	**1**
**c.14928C>G**	F4976L	MH 65 CCD	**2**	**1**	**0**	**0**	**0**	**1**	**0**	**0**	**0**
**c.7073T>C**	I2358T	MH 65 CCD	**2**	**2**	**0**	**0**	**0**	**0**	**0**	**0**	**0**
**c.742G>A**	G248R	MH 69	**1**	**1**	**0**	**0**	**0**	**0**	**0**	**0**	**0**
**c.10616G>A**	R3539H	MH 71	3	1							2
	R3539H	MH 242	1			1					
			**4**	**1**	**0**	**1**	**0**	**0**	**0**	**0**	**2**
**c.11723A>T**	N3908I	MH 73	**6**	**3**	**0**	**0**	**1**	**0**	**0**	**0**	**2**
**c.7355G>C**	R2452P	MH 77	**3**	**0**	**0**	**0**	**0**	**2**	**0**	**0**	**1**
**c.7361G>A**	R2454H	MH 89 CCD	**2**	**0**	**0**	**0**	**0**	**0**	**0**	**1**	**1**
**c.1201C>T**	R401C	MH 152	**3**	**2**	**0**	**0**	**0**	**0**	**0**	**0**	**1**
**c.1024G>A**	E342K	MH 153	**3**	**3**	**0**	**0**	**0**	**0**	**0**	**0**	**0**
**c.1021G>A, c.1021G>C**	G341R	MH 168	**1**	**1**	**0**	**0**	**0**	**0**	**0**	**0**	**0**
**c.6488G>A**	R2163H	MH 224	**1**	**1**	**0**	**0**	**0**	**0**	**0**	**0**	**1**
**c.3257C>T**	R1086H	MH 2	10	2		1	1	2		1	3
	R1086H	MH 197	1					1			
			**11**	**2**	**0**	**1**	**1**	**3**	**0**	**1**	**3**
**c.520C>T**	R174W	MH 104	3	1				1			1
			**3**	**1**	**0**	**0**	**0**	**1**	**0**	**0**	**1**
