# Supplementary material for: Relevance of pathogenicity prediction tools in human RYR1 variants of unknown significance
Source: Sci Rep. 2021 Feb 9;11:3445. doi: 10.1038/s41598-021-82024-7 (PMC7873245; doi:10.1038/s41598-021-82024-7)
Supplement: Supplementary file 2 — Supplementary Information 2. [file 41598_2021_82024_MOESM2_ESM.pdf]

Variant negativ individuals

Variant	Code-Nr.	DX	Halothan		Coffeine		Age	Gender
			Threshold (mM)	Contraction (mM)	Threshold (mM)	Contraction (mM)		
R614C	MH 1	MHS	1.00	N/A	2.00	2.00	49	male
R614C	MH 86	MHS	0.50	18.00	2.00	11.00	64	male
R614C	MH 165	MHS	2.00	3.80	2.00	10.00	17	male
R614C	MH 67	MHSc	3.00	N/A	1.00	3.00	72	male
R614C	MH 86	MHSc	5.00	17.00	1.00	21.00	13	male
R614C	MH 18	MHN	6.00	5.00	4.00	6.70	52	male
R614C	MH 18	MHN	6.00	22.00	4.00	21.00	63	male
R614C	MH 31	MHN	4.00	10.50	4.00	9.10	24	female
R614C	MH 31	MHN	4.00	7.50	4.00	1.80	6	female
R614C	MH 76	MHN	6.00	20.00	3.00	15.00	54	female
R614C	MH 119	MHN	5.00	5.40	5.40	32.00	47	male
R614C	MH 119	MHN	5.00	27.00	4.00	13.00	13	female
R614C	MH 165	MHN	4.00	30.30	4.00	21.20	57	male
R614C	MH 181	MHN	5.00	18.00	4.00	19.40	64	male
R614C	MH 181	MHN	3.00	16.70	3.00	12.80	19	male
R614C	MH 86	MHN	4.00	8.80	5.00	2.00	44	female
R614C	MH 86	MHN	5.00	12.00	3.00	10.00	39	female
G2434R	MH 14	MHS	1.00	17.00	1.00	13.00	26	female
G2434R	MH 14	MHS	1.00	13.00	2.00	10.00	27	female
G2434R	MH 90	MHS	2.00	17.00	1.50	9.30	8	male
G2434R	MH 90	MHS	2.00	13.00	2.00	19.40	55	male
G2434R	MH 102	MHS	2.00	187.00	2.00	165.00	45	male
G2434R	MH 129	MHS	2.00	8.70	2.00	8.50	4	male
G2434R	MH 241	MHS	0.50	288.00	0.50	295.00	2	female
G2434R	MH 241	MHS	0.50	172.00	1.00	149.00	5	female
G2434R	MH 102	MHSh	2.00	149.00	3.00	101.00	12	female
G2434R	MH 102	MHSh	1.00	119.00	4.00	61.00	27	female
G2434R	MH 102	MHSh	2.00	45.00	3.00	99.00	34	male
G2434R	MH 194	MHSh	2.00	5.40	3.00	9.70	2	male
G2434R	MH 8	MHN	6.00	8.80	3.00	9.80	37	male
G2434R	MH 14	MHN	2.00	3.00	2.00	1.00	27	male
G2434R	MH 14	MHN	N/A	N/A	4.00	0.60	14	female
G2434R	MH 90	MHN	5.00	7.40	3.00	12.00	58	male
G2434R	MH 90	MHN	6.00	13.00	4.00	15.00	43	male
G2434R	MH 90	MHN	5.00	13.00	4.00	11.40	84	female
G2434R	MH 90	MHN	5.00	8.50	3.00	8.80	16	male
G2434R	MH 90	MHN	5.00	21.90	3.00	11.70	33	female
G2434R	MH 102	MHN	6.00	76.00	3.00	159.00	50	male
G2434R	MH 109	MHN	5.00	155.00	4.00	112.00	18	male
G2434R	MH 191	MHN	6.00	5.00	4.00	4.40	31	male
G2434R	MH 194	MHN	5.00	14.00	3.00	4.10	37	female
G2434R	MH 194	MHN	3.00	9.50	3.00	17.70	40	male
G2434R	MH 210	MHN	3.00	11.70	3.00	21.30	37	female
G2434R	MH 210	MHN	5.00	214.00	3.00	120.00	37	male
R2336H	MH 52	MHS	1.00	7.00	1.00	13.00	35	male
R2336H	MH 85	MHS	1.00	23.00	1.50	16.00	66	male
R2336H	MH 85	MHSh	1.00	20.20	3.00	11.80	24	male
R2336H	MH 85	MHSh	1.00	10.00	4.00	12.00	29	female
R2336H	MH 71	MHN	4.00	N/A	3.00	10.80	13	male
R2336H	MH 71	MHN	4.00	6.00	4.00	10.40	38	female
R2336H	MH 71	MHN	4.00	8.00	4.00	9.00	11	male
R2336H	MH 71	MHN	5.00	15.00	4.00	13.00	41	male
R2336H	MH 71	MHN	4.00	N/A	3.00	0.80	9	female
R2336H	MH 71	MHN	4.00	N/A	4.00	0.60	63	female
R2336H	MH 85	MHN	5.00	9.30	4.00	12.00	60	male
R2336H	MH 85	MHN	5.00	7.90	3.00	16.00	27	female
R2336H	MH 85	MHN	2.00	14.90	3.00	9.90	59	male
R2336H	MH 85	MHN	5.00	13.20	3.00	7.60	67	female
R1667H	MH 90	MHN	4.00	7.40	3.00	12.90	58	male
R1667H	MH 90	MHN	4.00	13.00	4.00	20.30	55	male
R1667H	MH 90	MHN	4.00	8.50	4.00	11.40	33	female
R1667H	MH 90	MHN	4.00	2.10	3.00	8.80	17	female
R1667H	MH 90	MHN	6.00	13.00	3.00	11.00	30	male
R44C	MH 3	MHS	1.00	4.00	1.50	N/A	34	male
R44C	MH 3	MHS	2.00	39.80	2.00	15.40	41	female
R44C	MH 3	MHS	1.00	2.00	2.00	1.00	13	male
R44C	MH 3	MHS	0.50	391.00	1.00	679.00	55	male
R44C	MH 3	MHS	1.00	143.00	0.50	359.00	44	female
T2206M	MH 83	MHS	2.00	18.20	2.00	36.60	27	male
T2206M	MH 83	MHS	1.00	41.00	1.50	15.50	23	male
T2206M	MH 83	MHSc	5.00	12.00	1.50	11.00	58	male
T2206M	MH 56	MHSh	1.00	21.00	3.00	9.70	58	male
T2206M	MH 83	MHN	5.00	5.10	4.00	12.10	26	male
T2206M	MH 83	MHN	5.00	9.90	4.00	13.00	40	male
T2206M	MH 83	MHN	5.00	16.90	3.00	24.30	46	male
T2206R	MH 33	MHS	2.00	3.00	2.00	1.20	13	male
T2206R	MH 33	MHSH	1.00	2.00	3.00	2.00	9	male
T2206R	MH 33	MHN	6.00	1.00	3.00	1.00	16	male
T2206R	MH 33	MHN	6.00	N/A	3.00	1.20	51	female
T2206R	MH 33	MHN	3.00	N/A	3.00	1.10	13	female
H4833Y	MH 2	MHS	1.00	23.00	2.00	10.80	22	female
H4833Y	MH 2	MHSc	6.00	8.20	1.25	18.50	63	male
H4833Y	MH 2	MHN	6.00	11.40	4.00	8.80	63	male
H4833Y	MH 2	MHN	6.00	12.00	4.00	11.00	62	male
H4833Y	MH 2	MHN	6.00	2.00	3.00	1.00	33	male
H4833Y	MH 2	MHN	3.00	149.00	3.00	115.00	48	male
N2342S	MH 85	MHS	1.00	23.00	1.50	11.00	29	female
N2342S	MH 85	MHS	1.00	23.00	1.00	11.40	59	male
N2342S	MH 85	MHS	1.00	15.00	2.00	23.00	44	female
N2342S	MH 85	MHN	5.00	13.20	3.00	9.60	58	female
N2342S	MH 85	MHN	0.50	55.40	0.50	27.00	37	male
G2375A	MH 51	MHS	0.50	20.00	1.00	19.00	43	female
R2163C	MH 63	MHN	5.00	15.80	5.00	47.00	29	male
F4976L	MH 65	MHN	0.50	16.00	1.50	23.00	19	male
R3559H	MH 71	MHN	6.00	8.00	4.00	9.00	14	male
R3559H	MH 71	MHN	6.00	15.00	4.00	13.00	12	female
N3908I	MH 73	MHN	4.00	2.50	4.00	15.00	6	male
N3908I	MH 73	MHN	4.00	2.50	4.00	8.80	17	male
R2452P	MH 77	MHS	2.00	12.00	2.00	16.00	29	female
R2452P	MH 77	MHS	2.00	27.00	2.00	14.00	56	female
R2452P	MH 77	MHN	6.00	11.00	4.00	8.00	37	female
R2454H	MH 89	MHSh	2.00	14.00	4.00	15.00	50	male
R2454H	MH 89	MHN	5.00	2.50	3.00	7.00	21	male
R401C	MH 152	MHN	6.00	25.00	3.00	34.60	61	male
R1086H	MH 2	MHS	6.00	2.60	2.00	3.40	61	female
R1086H	MH 2	MHS	1.50	N/A	1.50	34.00	63	female
R1086H	MH 2	MHSh	2.00	253.00	4.00	14.00	22	female
R1086H	MH 2	MHN	4.00	11.00	32.00	10.00	39	male
R1086H	MH 2	MHN	6.00	1.00	5.00	N/A	26	male
R1086H	MH 2	MHN	2.50	12.50	32.00	N/A	66	male
R1086H	MH 197	MHS	1.00	16.90	2.00	12.80	22	female
R174W	MH 104	MHN	5.00	N/A	3.00	12.40	43	female
R174W	MH 104	MHS	2.00	36.00	2.00	27.00	44	male

Variant positiv individuals

Variant	Code-Nr.	DX	Halothan		Coffeine		Age	Gender
			Threshold (mM)	Contraction (mM)	Threshold (mM)	Contraction (mM)		
R614C	MH 1	MHS	0.50	N/A	1.50	2.20	71	female
R614C	MH 17	MHS	1.00	0.80	2.00	4.90	32	male
R614C	MH 17	MHS	0.50	12.70	1.50	3.00	59	male
R614C	MH 18	MHS	0.50	99.90	0.50	28.00	22	male
R614C	MH 31	MHS	1.50	5.50	2.00	2.00	38	female
R614C	MH 49	MHS	0.50	18.20	1.00	11.00	41	male
R614C	MH 62	MHS	1.00	3.00	1.50	8.00	28	female
R614C	MH 62	MHS	1.00	30.00	2.00	7.80	12	female
R614C	MH 67	MHS	2.00	25.00	2.00	9.60	51	male
R614C	MH 76	MHS	1.00	12.00	2.00	12.00	31	female
R614C	MH 76	MHS	1.00	35.00	1.00	40.00	64	female
R614C	MH 86	MHS	0.50	24.00	0.50	23.00	16	male
R614C	MH 86	MHS	2.00	15.00	1.50	22.00	59	male
R614C	MH 86	MHS	1.00	31.00	1.50	18.00	49	female
R614C	MH 86	MHS	1.00	30.00	1.50	23.00	16	female
R614C	MH 86	MHS	1.00	31.00	1.50	23.00	39	female
R614C	MH 86	MHS	2.00	15.00	2.00	17.00	21	male
R614C	MH 119	MHS	0.50	24.00	2.00	20.00	42	female
R614C	MH 119	MHS	1.00	47.00	1.50	11.90	29	male
R614C	MH 119	MHS	1.00	15.00	2.00	6.30	47	female
R614C	MH 165	MHS	1.00	21.00	2.00	13.60	38	female
R614C	MH 181	MHS	0.50	36.70	0.50	73.00	6	male
R614C	MH 249	MHS	1.00	225.00	1.50	320.00	39	female
R614C	MH 72	MHSh	1.00	10.90	3.00	7.00	73	female
R614C	MH 76	MHSh	1.00	14.00	3.00	15.00	31	female
R614C	MH 165	MHSh	2.00	3.80	4.00	20.80	19	female
R614L	MH 38*	MHS	0.50	99.90	0.50	12.00	26	male
G2434R	MH 8	MHS	1.00	7.00	1.75	1.50	78	male
G2434R	MH 14	MHS	1.00	14.80	1.50	6.40	45	male
G2434R	MH 14	MHS	1.00	28.00	1.00	12.00	42	male
G2434R	MH 14	MHS	1.00	132.00	1.50	135.00	42	female
G2434R	MH 14	MHS	1.00	193.00	1.50	119.00	32	male
G2434R	MH 29	MHS	1.00	1.90	2.00	0.85	34	male
G2434R	MH 29	MHS	1.00	21.00	1.50	20.00	32	male
G2434R	MH 29	MHS	0.50	21.50	1.00	26.40	23	female
G2434R	MH 29	MHS	1.00	14.90	1.00	73.00	37	male
G2434R	MH 29	MHS	1.00	10.00	1.50	8.00	68	male
G2434R	MH 29	MHS	1.00	0.75	1.00	31.00	10	male
G2434R	MH 29	MHS	2.00	13.00	2.00	12.00	18	female
G2434R	MH 29	MHS	1.00	25.00	1.50	15.00	63	female
G2434R	MH 29	MHS	1.00	141.00	2.00	114.00	69	male
G2434R	MH 29	MHS	1.00	26.00	1.50	12.00	79	female
G2434R	MH 29	MHS	2.00	8.80	2.00	15.00	67	female
G2434R	MH 102	MHS	0.50	254.00	1.50	227.00	38	male
G2434R	MH 102	MHS	0.50	272.00	1.00	257.00	34	male
G2434R	MH 102	MHS	1.00	230.00	1.50	193.00	20	male
G2434R	MH 102	MHS	0.50	494.00	1.00	287.00	14	female
G2434R	MH 102	MHS	0.50	279.00	0.50	245.00	22	female
G2434R	MH 102	MHS	1.00	272.00	1.50	319.00	38	male
G2434R	MH 102	MHS	0.50	241.00	1.00	190.00	37	female
G2434R	MH 106	MHS	1.00	283.00	1.50	177.00	10	female
G2434R	MH 109	MHS	1.00	225.00	2.00	210.00	46	female
G2434R	MH 129	MHS	1.00	29.00	1.50	33.00	29	female
G2434R	MH 129	MHS	0.50	40.00	1.00	19.00	9	male
G2434R	MH 191	MHS	1.00	7.60	1.50	14.00	21	female
G2434R	MH 191	MHS	1.00	22.00	1.50	14.00	8	female
G2434R	MH 191	MHS	0.50	48.00	1.00	22.00	11	female
G2434R	MH 194	MHS	1.00	28.00	1.50	15.50	5	female
G2434R	MH 194	MHS	0.50	191.00	1.50	230.00	12	male
G2434R	MH 199	MHS	0.50	25.20	0.50	26.30	13	female
G2434R	MH 199	MHS	1.00	16.90	0.50	19.00	38	male
G2434R	MH 210	MHS	2.00	10.50	2.00	14.00	13	male
G2434R	MH 215	MHS	0.50	33.00	1.00	23.00	32	male
G2434R	MH 241	MHS	1.00	213.00	2.00	186.00	28	female
G2434R	MH 29	MHSh	1.00	1.50	1.50	15.00	77	female
G2434R	MH 22	MHSh	2.00	3.00	3.00	1.00	21	female
G2434R	MH 29	MHN	5.00	2.50	4.00	13.20	33	male
R2336H	MH 71	MHS	1.00	13.00	1.50	8.30	42	male
R2336H	MH 71	MHS	2.00	6.40	1.00	4.00	70	male
R2336H	MH 71	MHS	1.00	99.00	1.00	99.80	12	male
R2336H	MH 85	MHS	0.50	12.00	0.50	27.00	19	male
R2336H	MH 85	MHS	1.00	15.00	2.00	23.00	12	male
R2336H	MH 71	MHS	2.00	98.00	2.00	99.80	7	male
R2336H	MH 85	MHS	1.00	20.00	12.00	11.80	61	male
R1667H	MH 90	MHS	2.00	19.90	2.00	19.60	16	male
R1667H	MH 90	MHS	2.00	13.20	2.00	19.60	8	male
R44C	MH 3	MHS	0.50	281.00	0.50	320.00	52	male
R44C	MH 3	MHS	0.50	176.00	1.00	277.00	35	female
R44C	MH 3	MHS	0.50	255.00	1.00	225.00	36	male
R44C	MH 3	MHS	0.50	2.00	1.00	12.80	13	male
T2206M	MH 56	MHS	0.50	7.00	0.50	3.00	23	male
T2206M	MH 56	MHS	0.50	14.00	1.00	13.00	53	female
T2206M	MH 56*	MHS	0.50	N/A	1.00	298.00	53	male
T2206M	MH 126	MHS	1.00	19.20	1.50	22.70	39	male
T2206M	MH 126	MHS	0.50	20.30	1.00	22.30	62	female
T2206M	MH 141*	MHS	0.50	452.00	0.50	338.00	39	male
T2206M	MH 83	MHSh	0.50	22.00	1.00	10.00	55	female
T2206R	MH 33	MHS	0.50	10.20	0.50	32.00	43	male
T2206R	MH 33	MHS	0.50	12.00	0.50	8.60	13	female
H4833Y	MH 2	MHS	1.50	N/A	1.50	3.20	37	male
R2454C	MH 5	MHS	0.50	9.90	1.00	12.50	25	male

Variant positiv individuals | MHS

	Halothan		Coffeine	
	Threshold (mM)	Contraction (mM)	Threshold (mM)	Contraction (mM)
Mean	0.97	64.57	1.52	60.96
Standard deviation	0.58	40.05	0.82	43.37

Variant positiv individuals | MHN

	Halothan		Coffeine	
	Threshold (mM)	Contraction (mM)	Threshold (mM)	Contraction (mM)
Mean	4.00	7.11	3.33	11.54
Standard deviation	1.26	4.17	0.52	5.52

Variant negativ individuals | MHN

	Halothan		Coffeine	
	Threshold (mM)	Contraction (mM)	Threshold (mM)	Contraction (mM)
Mean	4.61	21.01	4.33	19.08
Standard deviation	1.40	36.15	4.95	29.11

Variant negativ individuals | MHS

	Halothan		Coffeine	
	Threshold (mM)	Contraction (mM)	Threshold (mM)	Contraction (mM)
Mean	1.74	53.11	1.97	53.33
Standard deviation	1.35	80.46	37.38	119.94
