# Supplementary material for: Relevance of pathogenicity prediction tools in human RYR1 variants of unknown significance
Source: Sci Rep. 2021 Feb 9;11:3445. doi: 10.1038/s41598-021-82024-7 (PMC7873245; doi:10.1038/s41598-021-82024-7)
Supplement: Supplementary file 3 — Supplementary captions. [file 41598_2021_82024_MOESM3_ESM.docx]

Supplement 1: Comparison of genetic screening vs. in vitro contracture test.

Classification was in accordance to the European Malignant Hyperthermia Group Guidelines as follows:

Malignant hyperthermia susceptible halothane (MHSh): a halothane threshold concentration of 0.44 mmol L^-1^ or less in one halothane test and a caffeine threshold at a caffeine concentration of 3 mmol L^-1^ or more in all caffeine tests.

Malignant hyperthermia susceptible caffeine (MHSc): a caffeine threshold at a caffeine concentration of 2.0 mmol L^-1^ or less and a halothane threshold concentration above 0.44 mmol L^-1^ in all halothane tests.

Malignant hyperthermia susceptible halothane and caffeine (MSHhc): a caffeine threshold at a caffeine concentration of 2.0 mmol L^-1^ or less, in at least one caffeine test, and a halothane threshold concentration at 0.44 mmol L^-1^ or less, in at least one halothane test.

All malignant hyperthermia susceptible positive individuals including malignant hyperthermia susceptibility for only halothane (MHSh) or caffeine (MHSc) were treated as malignant hyperthermia susceptible.

Non Malignant hyperthermia susceptible (MHN): a caffeine threshold at a caffeine concentration of 3 mmol L^-1^ or more in all caffeine tests and a halothane threshold concentration above 0.44 mmol L^-1^ in all halothane tests.

Variant negative individuals were family members of variant positive individuals.

Supplement 2: In vitro contracture test results of each Individual included in the study
